# Microgametophyte Development in *Cannabis sativa* L. and First Androgenesis Induction Through Microspore Embryogenesis

**DOI:** 10.3389/fpls.2021.669424

**Published:** 2021-05-25

**Authors:** Alberto Galán-Ávila, Edgar García-Fortea, Jaime Prohens, Francisco Javier Herraiz

**Affiliations:** ^1^Ploidy and Genomics S.L., Centro Europeo de Empresas Innovadoras de Valencia, Parc Tecnològic, Valencia, Spain; ^2^Instituto Universitario de Conservación y Mejora de la Agrodiversidad Valenciana, Universitat Politècnica de València, Valencia, Spain

**Keywords:** amyloplast, cold-shock bud pretreatment, double haploids, microsporogenesis, microgametogenesis, pollen embryogenesis

## Abstract

Development of double haploids is an elusive current breeding objective in *Cannabis sativa* L. We have studied the whole process of anther and pollen grain formation during meiosis, microsporogenesis, and microgametogenesis and correlated the different microgametophyte developmental stages with bud length in plants from varieties USO31 and Finola. We also studied microspore and pollen amyloplast content and studied the effect of a cold pretreatment to excised buds prior to microspore *in vitro* culture. Up to 476,903 microspores and pollen grains per male flower, with *in vivo* microspore viability rates from 53.71 to 70.88% were found. A high uniformity in the developmental stage of microspores and pollen grains contained in anthers was observed, and this allowed the identification of bud length intervals containing mostly vacuolate microspores and young bi-cellular pollen grains. The starch presence in *C. sativa* microspores and pollen grains follows a similar pattern to that observed in species recalcitrant to androgenesis. Although at a low frequency, cold-shock pretreatment applied on buds can deviate the naturally occurring gametophytic pathway toward an embryogenic development. This represents the first report concerning androgenesis induction in *C. sativa*, which lays the foundations for double haploid research in this species.

## Introduction

*Cannabis sativa* L. is a multipurpose crop used by humans since at least 10,000 years ago (Abel, 1980). It is an allogamous and anemophilous species, which includes short and neutral-day varieties with androecious, gynoecious and monoecious specimens. Nowadays, this species is gaining increasing attention due to its medical applications (Abrams, 2018; Urits et al., 2019). Biologically active terpenophenolic metabolites known as cannabinoids are the main compounds responsible for the pharmacological properties of *C. sativa*. The allogamous nature of this species is translated into its inherent genetic and phenotypic heterogeneity, which results in reduced uniformity for food, fiber, or medical applications (Andre et al., 2016; Onofri and Mandolino, 2017). Regarding floral biology of *C. sativa*, it has been widely studied from different perspectives. Anatomy of male and female flower is briefly described in several publications (Reed, 1914; Miller, 1970; Zhou and Bartholomew, 2003; United Nations Office on Drugs and Crime (UNODC), 2009; Raman et al., 2017), and the genetic basis of sexual expression in cannabis has been elucidated (Hirata, 1927; Faux et al., 2014). DNA markers have also been reported as linked to *Cannabis* sex expression (Mandolino et al., 1999; Toth et al., 2020). Sex-reversal through induction of fertile male flowers on female plants is routine (Ram and Sett, 1982). Some detailed microscope studies of the cannabis female flower are also available (Hammond and Mahlberg, 1973; Spitzer-Rimon et al., 2019; Livingston et al., 2020).

Conversely, despite the key role that the androecium plays in important traits such as crop yield (Frankel and Galun, 2012; Pereira and Coimbra, 2019), especially through the male gametophyte, there is a lack of detailed studies on it in *C. sativa*. Most of the studies have been focused mainly in the process of meiosis carried out in the microsporangium (McPhee, 1924; Heslop-Harrison, 1966; Asanova, 2002), in the tapetum anther layer (Heslop-Harrison, 1962, 1971), or in the exine characterization of the mature pollen grain (Bradley, 1958; Punt and Malotaux, 1984).

Beyond the influence that the pollen grain has in traditional breeding and taxonomy, it takes exclusive prominence in androgenesis. Through this technique, it is possible to obtain 100% homozygous inbred lines in only one *in vitro* generation, thus allowing for fixation of traits and accelerating cultivars development. These plants are derived from a haploid nucleus of male origin and after spontaneous or induced chromosome doubling, double haploids are obtained. By means of hybridization of these pure lines, it is possible to exploit the hybrid vigor, obtaining high yielding and uniform F1 hybrid material. One of the routes that leads to androgenesis is microspore embryogenesis, by which the microspore deviates from its original gametophytic fate and it is reprogrammed to a new pathway of embryogenic development. Among the most relevant factors affecting microspore embryogenesis, is the microspore and pollen stage of development. It is widely accepted how vacuolate microspores and young bi-cellular pollen grains are more sensitive to the androgenic induction (Maheshwari et al., 1980; Dunwell, 2010; Dwivedi et al., 2015; Canonge et al., 2020). On the other hand, it has been demonstrated in different species how microspore and pollen stage of development can be correlated with some features of the flower, as is the case of bud length, pedicel length, anther length and petal to anther ratio in *Brassica napus* L. (Pechan and Keller, 1988), bud length and perianth morphological markers in *Solanum lycopersicum* L. (Brukhin et al., 2003), pigmentation degree of anthers (Kim et al., 2004) and calyx-corolla ratio (Bárány et al., 2005) in *Capsicum annuum* L., or more recently, flower bud size in *Stevia rebaudiana* Bertoni (Uskutoǧlu et al., 2019), and bud length, anther color, and filament length in *Opuntia ficus-indica* L. Mill (Bouamama-Gzara et al., 2020).

Furthermore, stress treatments are also described as highly relevant on microspore embryogenesis (Nitsch and Norreel, 1973a; Maheshwari et al., 1980; Touraev et al., 1997; Maraschin et al., 2005; Murovec and Bohanec, 2012; Dwivedi et al., 2015; Testillano, 2019). Different physical and chemical treatments, when applied to plants, inflorescences, flower buds, anthers or isolated microspores, can decisively promote the deflection of the gametophytic developmental pathway of microspores and pollen grains toward a sporophytic development (Shariatpanahi et al., 2006). Among the most popular stress treatments, cold-shock is the most frequently employed to promote microspore embryogenesis in a wide range of species. Its effect on microspore embryogenesis includes cytoskeletal organization disruption, reorganizing microspore and pollen-specific microtubule network thus blocking gametophytic division and promoting sporophytic development (Zhao et al., 2003). Other works mentioned its possible effect inhibiting the formation of starch grains in *Datura* proplastids and in pollen from *Hordeum vulgare* L. (Sangwan and Sangwan-Norreel, 1987). It has also been described how low temperature activates Ca^2+^ pathways and elicits an increase in cytosolic free calcium levels in microspores (Zoriniants et al., 2005; Żur et al., 2008), which could lead to increase protein phosphorylation events related with cell division and microspore embryogenesis (Pauls et al., 2006). Additionally, as reviewed by Shariatpanahi et al. (2006), cold slows down degradation processes in the anther tissues, thus protecting microspores from toxic compounds released in the decaying anthers, and low temperatures stimulate the expression of two heat-shock proteins (HSP) genes which possibly can protect cells against chilling injuries. Depending on the species and the explant submitted to the stress pretreatment, a cold-shock can be applied from some days to several weeks at a temperature about 4–10°C (Shariatpanahi et al., 2006). In general, cold-shock can be considered as more effective in terms of embryogenically induced microspores when applied directly to the flower buds (Nitsch and Norreel, 1973b; Sunderland and Wildon, 1979; Maheshwari et al., 1980; Deswal, 2018).

Finally, as it plays a key role on nutrition and viability of microspores and pollen grains, physiology and metabolism of carbohydrates in the androecium must also be considered as determinant for microspore embryogenesis. Additionally, amyloplasts appear as a marker of irreversible cell differentiation in the microspore (Clément and Pacini, 2001). Starch deposition has also been associated with a drastic change in protein synthesis (Mandaron et al., 1990), which could suggest the expression of genes involved in the gametophytic pathway and the consequent loss of cellular totipotency. In androgenic species, starch accumulation in microspores and pollen grains starts at the late bi-cellular pollen stage while in the recalcitrant species, there is an early accumulation of starch during microsporogenesis with an increase during pollen maturation (Sangwan and Sangwan-Norreel, 1987).

As can be deducted from the existing bibliography, which is mainly focused on the female flower, there are still many aspects of androecium development in *C. sativa* species that have not yet been clarified. Since detailed studies concerning a precise description of the whole process of microspore and pollen grain formation lacked in the related literature, this work is focused on the development of a comprehensive characterization of meiosis, microsporogenesis and microgametogenesis in *C. sativa*. By means of light and fluorescence microscopy on the one hand, and scanning electron microscopy (SEM) and cryo-SEM on the other, special attention was paid to the parallel development of the different layers that compose the anther tissue, together with the ornamentation of the exine of microspores and pollen grains, *in vivo* microspore viability and the different nuclear features observed during cell cycle regulation throughout all stages of pollen formation. On the other hand, in order to develop an experimental microspore culture protocol to induce microspore embryogenesis in *C. sativa*, the correlation of the different developmental stages of microspores and pollen grains with bud length was studied. Furthermore, we also studied the androgenic potential of *C. sativa* through the microscopic analysis of the amyloplasts contained in anthers, microspores and pollen grains. Finally, the effect of a week-long cold pretreatment applied directly on excised buds before microspore culture was evaluated in terms of microspore viability, amyloplast content of microspores and development of multicellular structures of androgenic origin. Short and neutral-day varieties, together with androecious and monoecious specimens were used in our experiments. Additionally, due to their exclusive capability for cannabinoid synthesis and their influence on breeding of the species, also gynoecious specimens treated with silver thiosulphate anionic complex (STS) for sex-reversal were added to our experimental design. Our work provides new improvements and updates on the morphology and male floral biology of *C. sativa*, laying the foundations for the routine implementation of androgenesis in *C. sativa* breeding.

## Materials and Methods

### Plant Material and Growth Conditions

Staminate floral buds needed for all experiments were collected from androecious, monoecious and gynoecious plants from short-day variety USO31. Additionally, due to the marked dioecious character of the neutral-day variety Finola (which lacked monoecious specimens), only androecious and gynoecious individuals from this variety were used as donor plants. Seeds were germinated in pots (1 L) with fertilized commercial substrate composed of a mixture of black peat, granulated peat moss and perlite, with a pH value of 6 and a conductivity of 1 mS/cm. In order to induce the formation of male flowers in gynoecious individuals, 2 weeks after germination of the seeds, female plants were sprayed once a day (early in the morning), during five consecutive days, with an aqueous solution of silver thiosulphate anionic complex [(STS); 1 silver nitrate (AgNO_3_) and 8 sodium thiosulphate (Na_2_S_2_O_3_) w/w], as described by Ram and Sett (1982). After spraying, plants were kept in darkness for 1 h, and then returned to the growth chamber. Plants were grown under controlled environmental conditions at 25°C ± 1°C and 60% ± 1% relative humidity. During the whole cultivation process, photoperiod consisted of 12 h of light per day. Light was provided by Lumilight^®^ Led Grow Monster LPW-220 (LUMILIGHT LED GROW Ltd., Valencia, Spain), equipped with Light Emitting Diodes (LEDs) of 220W and a color temperature of 2,470K, which supplied 16,700 lumens and 546 μmol m^–2^ s^–1^. Once a day, plants were watered (75% tap water + 25% osmotized water) through drip-irrigation. Following this protocol, staminate floral buds from all evaluated phenotypes were collected approximately 30 days after seed germination. Plants employed in this study were grown under license for the cultivation of *C. sativa* for research purposes, issued by the Spanish Ministry of Health via Spanish Agency of Medicines and Health Products (Agencia Española de Medicamentos y Productos Sanitarios or AEMPS) to Ploidy and Genomics Ltd.

### Characterization of Nuclear Dynamics During Meiosis, Microsporogenesis and Microgametogenesis Through Light and Fluorescence Microscopy

Male floral buds of different sizes containing anthers in all developmental stages of meiosis, microsporogenesis and microgametogenesis were dissected, and two anthers per bud were separately placed in a glass slide with 10 μL of a 2.5 μg/ml solution of DAPI (4′, 6-diamidino-2-phenylindole) to stain nuclear DNA (Kapuscinski, 1995). Anthers were cut in thin sections with a scalpel and their content was exposed to the DAPI solution, while remaining somatic tissue was removed from the slide. Meiocytes, microspores and pollen grains contained in anthers were observed with a Carl Zeiss^®^ Axiovert.A1 (CARL ZEISS MICROSCOPY Ltd., Jena, Germany) inverted microscope equipped with epi-fluorescence excitation LED modules, and images were registered with a Carl Zeiss^®^ Axiocam 305 color (CARL ZEISS MICROSCOPY Ltd.). Measurements on images obtained with the microscope were carried out using ImageJ 1.53a (Schneider et al., 2012). The different developmental stages of meiocytes, microspores and pollen grains observed were classified as follows: microspore mother cell, meiocyte with two nuclei, meiocyte with four nuclei, tetrad, young microspore, mid microspore, vacuolate microspore, young bi-cellular pollen, mid bi-cellular pollen and mature tri-cellular pollen (Crang et al., 2018). For simplification and due to the absence of significant differences between them, young and mid microspores were merged into a single stage of development.

### Analysis of Exine Evolution by Means of Scanning Electron Microscopy (SEM)

With the aim of studying the ornamentation of the exine of microspores and pollen grains from *C. sativa*, in the first place, their respective stages of development were determined through DAPI staining of one of the anthers contained in each bud, as described above. To avoid mixing microspores and pollen grains at different stages of development, microspore isolation of each developmental stage was performed separately. For each stage, 5–10 buds exclusively containing anthers in a specific developmental stage were selected. The stages of development studied in this experiment ranged from tetrad until mature pollen (as mature tri-cellular pollen grains never composed exclusively the population of a pollen sac, but coexisted with mid bi-cellular pollen grains in the locule, anthers containing both stages of development were merged and classified in this experiment as mature pollen stage). Microspore extraction was carried out under cold conditions (4–8°C) by gentle squashing of the buds with the plunger of a sterile syringe. Microspores and pollen grains were isolated in MS liquid medium (Murashige and Skoog, 1962) by filtration through two layers of 40 μm nylon filter. Previously, MS liquid medium was sterilized by vacuum filtration through a 0.22 μm polyethersulfone (PES) membrane. Samples were washed with deionized water and centrifuged three times (5 min each) at 8°C and 110.7 *g* and, after supernatant was discarded, microspores were deposited in a 11 μm pore size filter bag and exposed to a formaldehyde-glutaraldehyde fixative as described by Karnovsky (1967). Then, samples were washed and kept in cacodylate buffer (0.025 M) at 4°C overnight. They were dehydrated through a series of ethanol concentrations in deionized water (70, 80, 95, 100%; 1 h each) and, subsequently, ethanol was replaced by CO_2_ and intracellular CO_2_ was evaporated at 34°C and 73.7 bar in a Leica Microsystems^®^ EM CPD300 (LEICA MICROSYSTEMS Ltd., Wetzlar, Germany) automatic critical point drying apparatus. Following this, samples were deposited on a support covered by double-sided tape and were platinum sputtered during 15 s prior to visualization in a Carl Zeiss^®^ ULTRA 55 (CARL ZEISS MICROSCOPY Ltd.) scanning electron microscope, with an electron acceleration of 2 kV and a working distance of 4≈8 mm. Images were taken with the software INCA from Oxford Instruments^®^ (OXFORD INSTRUMENTS Ltd., High Wycombe, United Kingdom). Measurements of the images obtained with the microscope were carried out using ImageJ 1.53a (Schneider et al., 2012). Terminology used to define the ornamentation of the exine was extracted from the glossary published by Laín (2004).

### Study of Anther Wall Formation Using Cryogenic Scanning Electron Microscopy (cryo-SEM)

In order to study the development of the different layers that compose the anther wall during its growth, staminate floral buds of different sizes containing anthers in all developmental stages of meiosis, microsporogenesis, and microgametogenesis were dissected. The stage of development of the microspores and pollen grains from one of the five anthers contained in each flower was determined with DAPI staining as described above. The four remaining anthers of each bud were frozen by immersion in slush nitrogen. Water sublimation of the samples was performed at a temperature of –90°C during 15 min in a Quorum Technologies^®^ PP3010 (QUORUM TECHNOLOGIES Ltd., Laughton, East Sussex, United Kingdom) cryo preparation system for SEM. After freeze-fracture of the anthers, samples were platinum sputtered during 60 s and observed with a Carl Zeiss^®^ ULTRA 55 (CARL ZEISS MICROSCOPY Ltd.) scanning electron microscope at a temperature ranging from –150 to –180°C, an electron acceleration of 2 kV and a working distance of 4≈8 mm. Images were taken with the software INCA from Oxford Instruments^®^ (OXFORD INSTRUMENTS Ltd.). Measurements of the images obtained with the microscope were carried out using ImageJ 1.53a (Schneider et al., 2012). Due to the absence of morphological differences between them, stages corresponding to meiocyte with two nuclei and meiocyte with four nuclei were merged together with microspore mother cell in a single meiotic stage of development. Moreover, as mature tri-cellular pollen grains never composed exclusively the population of a pollen sac, but coexisted with mid bi-cellular pollen grains in the locule, anthers containing both stages of development were merged and classified in this experiment as mature pollen stage.

### Histochemical Detection of Starch Through Light Microscopy

For starch recognition in anthers, meiocytes, microspores and pollen grains from *C. sativa*, buds of different sizes covering all stages of development were dissected and one of the five anthers contained in each flower, was placed in a glass slide and stained with DAPI as described above. After identification of its developmental stage, the four remaining anthers were fixed in Karnovsky solution as described above. Samples were kept in cacodylate buffer (0.025 M) at 4°C overnight. After that, samples were dehydrated through a series of ethanol concentrations in deionized water as previously described, and infiltrate and embedded in Technovit^®^ 7100 (KULZER Ltd., Wehrheim, Germany) acrylic resin, as specified by the manufacturer. Resin sections (1.5 μm) were cut with a glass knife using a Reichert - Jung^®^ (now: Leica Microsystems^®^) Ultracut E (LEICA MICROSYSTEMS Ltd., Wetzlar, Germany) ultramicrotome, collected on glass slides and exposed to Lugol^®^ (MERCK Inc., Darmstadt, Germany) solution during 5 min for iodine-starch complex staining and detection of amyloplasts contained in microspores and anthers. After rinsing in distilled water and drying, preparations were mounted in glycerol and observed in a Carl Zeiss^®^ Axiovert.A1 (CARL ZEISS MICROSCOPY Ltd.) inverted microscope. Histochemical detection of starch was also performed on excised male buds after 1 week long cold pretreatment at 4°C ± 1°C. Due to the absence of differences between them, stages corresponding to meiocyte with two nuclei and meiocyte with four nuclei were merged together with microspore mother cell in a single meiotic stage of development. Moreover, as mature tri-cellular pollen grains never composed exclusively the population of a pollen sac, but coexisted with mid bi-cellular pollen grains in the locule, both stages of development were merged and classified in this experiment as mature pollen developmental stage.

### Correlation of the Bud Length With the Stage of Development of Microspores and Pollen Grains

Staminate floral buds from androecious, monoecious, and gynoecious plants of short-day variety USO31, and from androecious and gynoecious plants of neutral-day variety Finola, were manually excised and grouped in one-millimeter length intervals ranging from 1.00 to 6.99 mm, covering the whole range of bud development. A minimum of 18 buds coming from at least three different plants were considered for each length interval studied. Buds were observed and dissected with an Optika^®^ SZN-6 (OPTIKA S.r.l., Ponteranica, Italy) laboratory stereo zoom microscope, and images were registered with an Optika^®^ C-HP (OPTIKA S.r.l.) digital camera adapted to the stereoscopic microscope, which allowed for live measurements. Bud measurement was carried out from the pedicel insertion point to the bud tip. As described above, to determine its stage of development and after flower dissection, one anther per bud was placed in a glass slide and its content was exposed to the DAPI solution prior to visualization in the microscope. A minimum of 200 randomly chosen meiocytes, microspores or pollen grains per anther preparation were counted, classifying them following the above described different developmental stages. For each bud length interval, the mean of each developmental stage was expressed as a percentage (±SE) relative to the total sample size of the interval. Stages corresponding to microspore mother cell, meiocyte with two nuclei and meiocyte with four nuclei were merged together in a single meiotic stage of development. As mature tri-cellular pollen grains were observed after anthesis, it was not possible to scale buds containing this stage, so they were excluded from this experiment.

### *In vitro* Microspore Culture Experiments

Male flowers from androecious, monoecious, and gynoecious plants of short-day variety USO31, and from androecious and gynoecious plants of neutral-day variety Finola were used for microspore embryogenesis experiments. In order to evaluate the effect of stress on microspore embryogenesis, we compared isolated microspore cultures coming from non-pretreated staminate buds on the one hand, and from excised male buds exposed to a week-long cold-shock at 4°C ± 1°C prior to *in vitro* culture on the other in terms of microspore viability, amyloplast content of microspores and development of multicellular structures of androgenic origin. Microspores coming from different phenotypes were isolated and cultured separately, and experiments were repeated three times. Each microspore culture replicate consisted of 18 mL which were distributed in 6 cm diameter plastic Petri dishes. Microspore density was adjusted to 40,000 microspores/mL, as described by Huang et al. (1990). For each microspore culture, 12 buds exclusively containing vacuolate microspores and young bi-cellular pollen grains coming from at least three different plants were surface sterilized by immersion in 20 g/L of NaClO with 0.1% (v/v) Tween 20 during 10 min, and finally washed three times in sterile water for about 1, 4, and 10 min each. Vacuolate microspores and pollen grains were isolated under cold (4–8°C) and aseptic conditions as described above. Filter-sterilized MS liquid medium containing 0.04 mg/L of kinetin (KIN) and 1.0 mg/L of indoleacetic acid (IAA) (Murashige and Skoog, 1962), was used for isolation and culture of microspores and pollen grains. The medium pH was adjusted to 5.8 with NaOH. After centrifugation and supernatant discarding, microspore density was calculated by using a hemocytometer counting slide (Neubauer improved cell counting chamber). Microspore cultures were kept in dark and grown under controlled environmental conditions at 25°C ± 1°C and 60% ± 1% of relative humidity. Viability measurement of microspores and pollen grains coming from both non-pretreated and cold-pretreated buds was carried out immediately after microspore extraction. Viability quantification was carried out through light and fluorescence microscopy. For this, isolated microspores and pollen grains were stained in a 0.4 M sucrose solution containing fluorescein diacetate (FDA) at a concentration of 4.8 μM, as described by Zottini et al. (1997). Data related to microspore density per flower bud and microspore *in vivo* viability obtained from this experiment were used for characterization of the different phenotypes evaluated in the present research. Furthermore, development of multicellular structures of androgenic origin was evaluated by means of light and fluorescence microscopy after staining with 10 μL of a 2.5 μg/ml solution of DAPI. Microspore cultures were examined just after *in vitro* culture establishment and every 3 weeks during a total period of 3 months with a Carl Zeiss^®^ Axiovert.A1 (CARL ZEISS MICROSCOPY Ltd.) inverted microscope equipped with epi-fluorescence excitation LED modules.

### Data Analyses

Parameters of the different phenotypes evaluated such as plant height, microspores per flower bud, and *in vivo* viability of microspores were statistically analyzed. In order to correlate the different developmental stages of microspores and pollen grains from *C. sativa* with the bud length, percentages of the different stages of development were statistically compared among the different length intervals established for each of the phenotypes evaluated. Additionally, in order to verify the correlation among pollen maturation and the different flower bud length intervals established, Spearman rank correlation coefficient was calculated after data pooling from all *C. sativa* varieties. Finally, viability of microspores from non-pretreated staminate buds on the one hand, and from male buds exposed to a week-long cold-shock at 4°C ± 1°C prior to *in vitro* culture on the other, were statistically compared. Independence among variables (Durbin–Watson test), homoscedasticity (Bartlett’s test for mean variance analysis or Fligner-Killeen median test), and normality (Shapiro–Wilk test) were evaluated for the data coming from the experiments and, depending on results, ANOVA parametric test followed by Fisher’s least significant difference (LSD) test (*p* < 0.05), or Kruskal–Wallis non-parametric test followed by pairwise Nemenyi test (*p* < 0.05), were used to statistically determine significant differences between levels of each factor evaluated. Statistical analysis was carried out using R software (R Core Team, 2019).

## Results

### Male Reproductive Anatomy of *C. sativa*, Microspore Density per Flower Bud and *in vivo* Viability of Microspores

**FIGURE 1:**
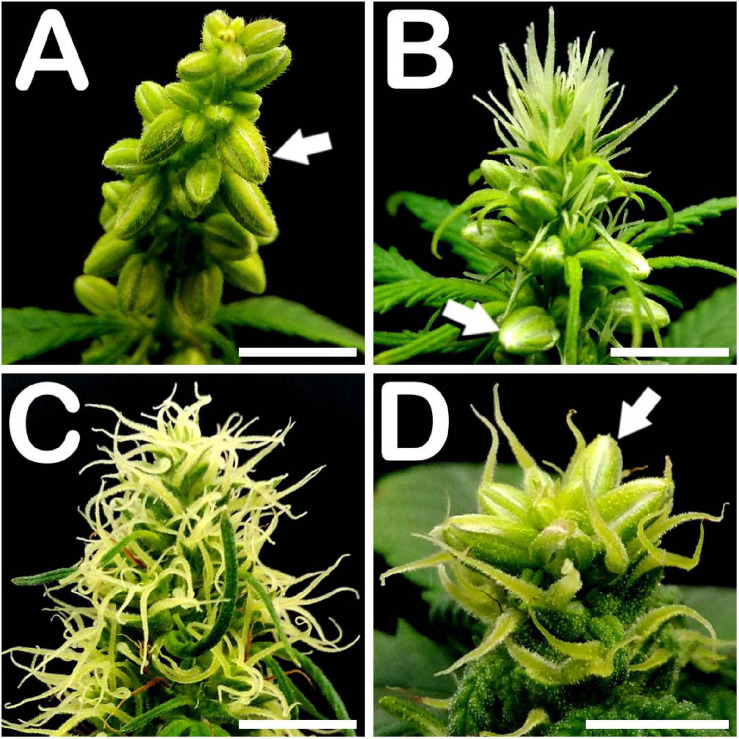
Floral apices of different *C. sativa* phenotypes. **(A)** Panicle from an androecious plant showing prominent production of staminate flowers: arrow points to a male flower bud. **(B)** Floral apex from a monoecious specimen fully covered by pistillate flowers on its apical section, and showing male floral buds in its middle section: arrow points to a staminate flower. **(C)** Apex from a gynoecious plant, showing multiple pistillate flowers. **(D)** STS treated gynoecious plant showing male bud formation on its apical section coexisting with pistillate flowers: arrow points to a staminate flower. Scale bars: 5 mm.

Approximately 30 days after seed germination, all phenotypes studied showed male flowers in all developmental stages of stamen formation, including anthesis. Androecious individuals showed a prominent production of staminate buds, mostly located on the main apical meristem of the plant and arranged in a panicle (Figure 1A). Regarding monoecious specimens, while the top of the apical meristem was fully covered by pistillate flowers, its middle section produced a high amount of male floral buds (Figure 1B). Finally, in contrast to what occurred with non-treated gynoecious plants, whose floral apices appeared fully covered by pistillate flowers shaping the female inflorescence of the plant (Figure 1C), STS treated gynoecious plants yielded a remarkable number of staminate flowers mainly situated on the top of the apical meristem (Figure 1D).

Statistically significant differences in plant height were detected among the different phenotypes evaluated (Table 1). Specifically, androecious specimens from neutral-day variety Finola were significantly taller than the other varieties tested, reaching a plant mean height of 35.18 cm just 30 days after germination of the seeds, while gynoecious plants had the lowest plant mean height of all the evaluated phenotypes with 17.38 cm (Table 1). Plants from short-day variety USO31 had a similar mean height for androecious, gynoecious, and monoecious specimens (Table 1).

**TABLE 1:** Evaluation of plant height, microspore and pollen density per flower bud and *in vivo* microspore and pollen viability in different phenotypes of *C. sativa*.

**Reproduction**	**Photoperiodism**	**Gender**	**Variety**	**Plant height (cm)**	**Microspores per flower bud**	**Microspore viability (%)**
Dioecious	Neutral-Day	♂	Finola	35.18^a^ ± 1.93	156,944^b^ ± 12,483	53.71^a^ ± 4.61
Dioecious	Neutral-Day	♀ (STS)	Finola	17.38^b^ ± 1.39	255,494^ab^ ± 25,668	59.71^a^ ± 4.07
Dioecious	Short-Day	♂	USO31	22.82^b^ ± 2.25	476,903^a^ ± 64,503	70.88^a^ ± 4.27
Dioecious	Short-Day	♀ (STS)	USO31	21.00^b^ ± 0.82	303,889^ab^ ± 32,961	60.49^a^ ± 1.45
Monoecious	Short-Day	♂ + ♀	USO31	19.06^b^ ± 0.86	471,865^a^ ± 83,205	65.87^a^ ± 3.64

*Means are expressed as a percentage (±SE) calculated from data coming from at least three replicates. Different letters among factors indicate significant differences between them (p < 0.05) according to non-parametric Kruskal–Wallis and pairwise Nemenyi tests. Not significant differences were detected among factors evaluated in “Microspore viability (%)” column according to parametric ANOVA test. ♂: “Only male flowers present in the plant”. ♀: “Only female flowers present in the plant”. ♂ + ♀: “Male and female flowers present in the plant”.*

Significant differences among phenotypes were also observed for microspores and pollen grains produced per flower bud (Table 1). In this case, short-day variety USO31 showed a higher capability for pollen production than neutral-day variety Finola. Androecious plants from USO31 yielded the highest microspore density of this experiment with 476,903 microspores and pollen grains per flower bud, followed by monoecious and gynoecious individuals with, respectively, 471,865 and 303,889 microspores per flower bud (Table 1). Gynoecious and androecious plants from neutral-day variety Finola displayed a lower efficiency in terms of pollen production with, respectively, 255,494 and 156,944 microspores per flower bud (Table 1).

No significant differences were observed among the different phenotypes evaluated for *in vivo* viability of microspores and pollen grains (Table 1). Microspore viability ranged from 53.71% for microspores and pollen grains from androecious plants of neutral-day variety Finola, to 70.88% for microspores and pollen grains coming from androecious specimens of short-day variety USO31 (Table 1). Viability of microspores and pollen grains from STS treated gynoecious plants from both USO31 and Finola varieties, achieved similar viability levels as those from androecious and monoecious specimens (Table 1).

### Cellular Characterization of the Different Developmental Stages of Meiosis, Microsporogenesis, and Microgametogenesis

**FIGURE 2:**
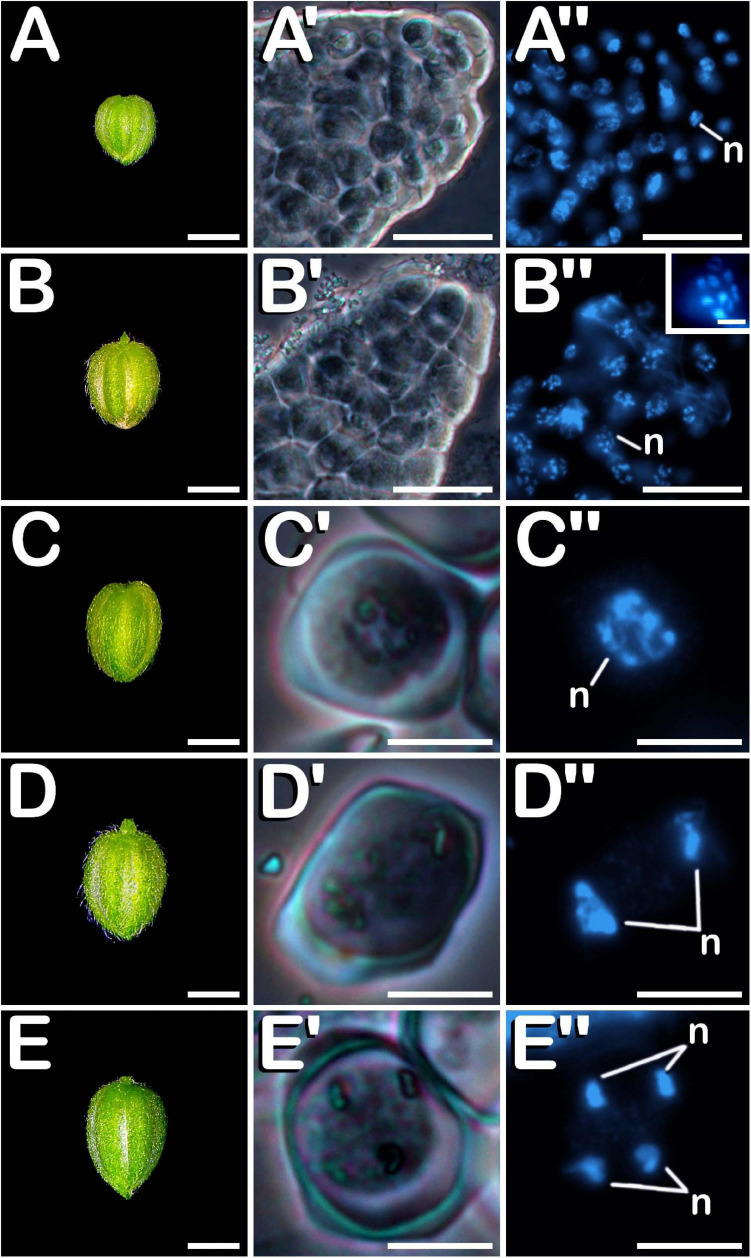
Meiosis development in *C. sativa*. The different developmental stages are described as follows: **(A–E)** *C. sativa* var. Finola (dioecious male) staminate flowers growing in size. **(A′,A″)** Compact mass of clustered microspore mother cells in prophase I. **(B′,B″)** Compact mass of clustered microspore mother cells in metaphase I and individual microspore mother cell showing 10 bivalents (inset in B″). **(C′,C″)** Isolated microspore mother cell. **(D′,D″)** Meiocyte with two nuclei in telophase I. **(E′,E″)** Meiocyte with four nuclei in telophase II. **(A′–E′)** Phase-contrast microscope images. **(A″–E″)** Fluorescent microscope images after DAPI staining. Scale bars **(A–E)**: 1 mm. Scale bars **(A′,B′)** and **(A″,B″)**: 40 μm. Scale bar (inset in **B″**): 5 μm. Scale bars **(C′–E′)** and **(C″–E″)**: 10 μm. n, nucleus.

Regardless of the anther maturity degree, all evaluated phenotypes presented highly uniform anthers containing microspores and pollen grains in a predominant developmental stage (with the only exception of mature tri-cellular pollen grains, which never composed exclusively the population of a pollen sac, but coexisted with mid bi-cellular pollen grains in the locule). This synchronized development was also observed among different anthers coming from the same bud, which enclosed microspores and pollen grains in the same stage of development. Microsporogenesis initiated when microspore mother cells (MMCs) proceeded through meiosis. Meiotic stages of development were present in buds ranging from 1.00 to 2.99 mm in the neutral-day variety Finola (Figures 2A–E), and from 1.00 to 3.99 mm in the short-day variety USO31. Pollen grain formation started from a compact mass of clustered MMCs (Figure 2A′) in which multiple nuclei were observed (Figure 2A″). This kind of cells were firmly attached one to each other (Figure 2B′), and underwent meiosis synchronously (Figure 2B″). Some nuclei exhibited 10 bivalents clearly differentiated and centrally located in the metaphase plate (inset in Figure 2B″). Isolated MMCs showed a regular polygonal shape, with a rounded cytoplasm encircled by a thick callose layer (Figure 2C′), and with a large nucleus located in the middle of the cytoplasm (Figure 2C″). After meiosis I, meiocytes with two nuclei were generated. As observed in MMC stage of development, they also showed a thick callose coat surrounding their rounded cytoplasm (Figure 2D′), although the main characteristic of this phase was the presence of two prominent nuclei located in the cytoplasm (Figure 2D″). There were no evidences of wall formation separating nuclei and cytoplasm at meiocyte with two nuclei stage of development. Successively, development of meiosis II gave rise to meiocytes with four nuclei, which still preserved their thick external callose layer and their rounded cytoplasm (Figure 2E′). They were characterized by the presence of four nuclei clearly distinguishable in their cytoplasm (Figure 2E″). As in the previous stage of development, no wall separating their nuclei and cytoplasm was observed. All these developmental stages of meiosis were common for the three types of plants and for the two varieties evaluated.

**FIGURE 3:**
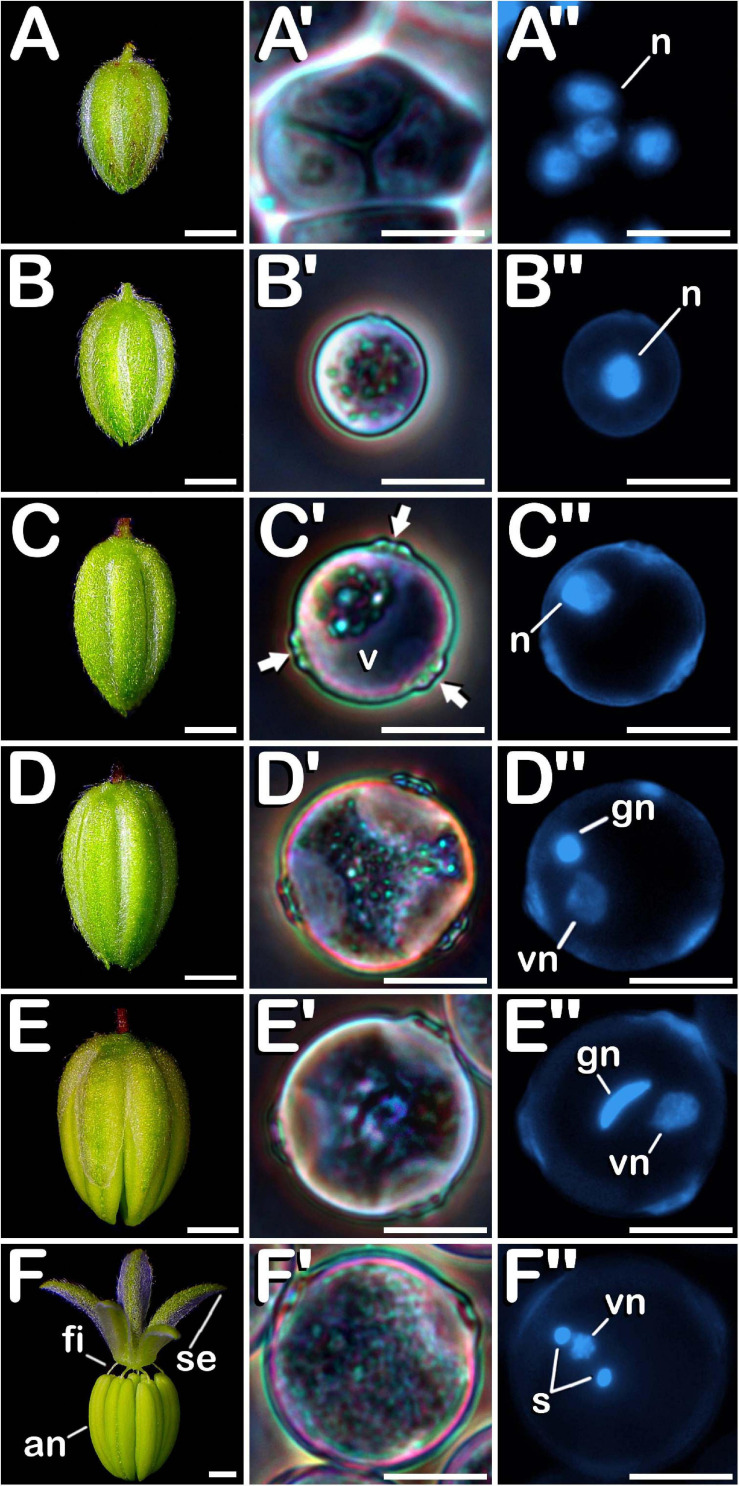
Microsporogenesis and microgametogenesis developed in *C. sativa*. The different developmental stages are described as follows: **(A–E)** *C. sativa* var. Finola (dioecious male) staminate flowers growing in size. **(F)** *C. sativa* male flower after anthesis. **(A′,A″)** Tetrad stage. **(B′,B″)** Young and mid microspore stage. **(C′,C″)** Vacuolate microspore stage: arrows highlight apertures. **(D′,D″)** Young bi-cellular pollen stage. **(E′,E″)** Mid bi-cellular pollen stage. **(F′,F″)** Mature tri-cellular pollen stage. **(A′–F′)** Phase-contrast microscope images. **(A″–F″)** Fluorescent microscope images after DAPI staining. Scale bars **(A–F)**: 1 mm; Scale bars **(A′–F′)** and **(A″–F″)**: 10 μm. n, nucleus; v, vacuole; vg, vegetative nucleus; gn, generative nucleus; an, anther; fi, filament; se, sepal; s, spermatids.

Post-meiotic stages were observed in buds ranging from 1.00 to 5.99 mm in the case of neutral-day variety Finola (Figures 3A–E), and from 2.00 to 5.99 mm in the case of short-day variety USO31. After meiosis, tetrads (Figure 3A′) were visualized. They were characterized by the presence of four haploid microspores of polygonal shape constricted in a tetrahedral disposition by a callose wall resembling the thick coat observed in MMC and more advanced meiotic developmental stages. The perspective of the nuclei observed in Figure 3A″ revealed the tetrahedral arrangement of microspores inside the tetrad. After degradation of the tetrad wall, the spherical young and mid microspores (Figure 3B′), with a prominent and centrally located nucleus (Figure 3B″), were released. At this phase, microspores reached a diameter of ≈12 μm. During their development, microspores went through a progressive vacuolation process, concluded with the formation of a large vacuole, main characteristic of vacuolate microspores (Figure 3C′). At this developmental stage, also an increase in exine thickness was observed, which allowed visualization of three prominent apertures distributed throughout the exine (arrows in Figure 3C′). The vacuole occupied most of the cytoplasm, displacing the nucleus toward the cell periphery (Figure 3C″). Commonly considered as one of the suitable developmental stages for the induction of microspore embryogenesis, vacuolate microspores were also characterized by an increase in volume compared with young and mid microspores. The diameter of vacuolate microspores was ≈17 μm. Subsequently, an asymmetric mitotic division of the nucleus was observed. This first pollen mitosis, defines the young bi-cellular pollen stage (Figure 3D′), determining the completion of microsporogenesis and the start of microgametogenesis. Noteworthy, together with vacuolate microspore, young bi-cellular pollen stage is usually defined as the most sensitive stage of development for androgenic induction in a wide range of species. Its main feature is the presence of two cells of different size and level of chromatin condensation in the cytoplasm. The vegetative cell grew and its chromatin appeared more dispersed, showing weaker fluorescence in comparison with the generative cell (Figure 3D″). A slight increase in volume was appreciated, with young pollen grains acquiring a diameter of ≈19 μm. In succession to these stages, mid bi-cellular pollen grains (Figure 3E′) were visualized whose diameter reached ≈21 μm, although the main difference with respect to the previous stage of development was observed by means of fluorescence microscopy. First, the vegetative cell migrated to the center of the pollen grain. After that, generative nucleus moved to the center, close to the vegetative nucleus and acquired a fusiform morphology (Figure 3E″) which finally defined the mid bi-cellular pollen stage. Until this phase, flower buds increased in size (Figures 3A–E) as more advanced stages of development were observed. However, only after anthesis (Figure 3F) and prior to the issuance of the pollen tube, mature tri-cellular pollen grains (Figure 3F′) were visualized. No significant variations in diameter were observed among mid bi-cellular and mature tri-cellular pollen stages of development. Mature tri-cellular pollen grains had a diameter of ≈23 μm. Nonetheless, an important nuclear event took place at this stage. Generative cell, initially inactive as revealed by a high degree of chromatin condensation, entered in cell cycle and underwent second pollen mitosis, which led to the formation of the two spermatid cells (Figure 3F″), typical from the mature tri-cellular pollen stage of development. It is remarkable how this stage never was exclusively present in a single loculus, always being visualized together with mid bi-cellular pollen grains. This developmental process including characteristics of each stage, was observed for both Finola and USO31 varieties, and also for androecious, monoecious and gynoecious plants. In all evaluated phenotypes, anthers coming from the same bud always showed the same developmental stage.

### Evolution of the Exine Throughout Microsporogenesis and Microgametogenesis

**FIGURE 4:**
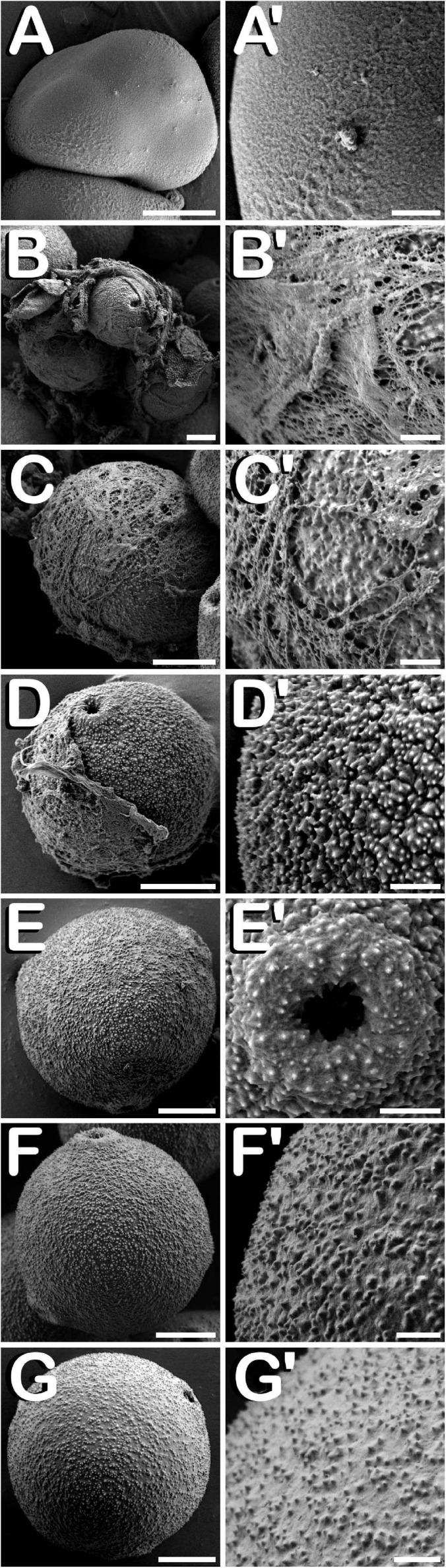
SEM images showing detailed development of the exine ornamentation during microsporogenesis and microgametogenesis in *C. sativa* var. USO31 (monoecious). The different developmental stages are described as follows: **(A–C,A′–C′)** Tetrad stage: detail of the callose layer covering the four enclosed microspores can be observed in **(A′)**; fissures and holes as a result of callose degradation can be seen in **(B′)**; microspore exine appears among callose fibers in **(C′)**. **(D,D′)** Young and mid microspore stage. **(E)** Vacuolate microspore stage. **(E′)** Detail of an aperture of a vacuolate microspore. **(F,F′)** Young bi-cellular pollen stage. **(G,G′)** Mature pollen stage. Scale bars **(A–G)**: 5 μm. Scale bars **(A′–G′)**: 1 μm.

The surface of microspores and pollen grains from tetrad until mature pollen stage of development was studied through SEM. In the early tetrad stage, a callose wall was fully covering the four microspores contained inside of the tetrad (Figure 4A). At this phase, the tetrad surface presented a regular and smooth texture (Figure 4A′). As microsporogenesis advanced, the callose coat was progressively degraded until the shape of the microspores contained in the tetrad was noticeable (Figure 4B). Deterioration of callose wall was characterized by the formation of fissures and holes along the entire tetrad surface (Figure 4B′), which lost its regular and smooth pattern, giving way to an irregular, disorganized and porous texture. Just before being released, in late tetrad stage, microspores presented a disordered surface of rough appearance and apertures were still covered by callose remnants (Figure 4C). At this phase, fissures and holes extended in size, which allowed observation of the microspore exine among residual callose fibers (Figure 4C′). Once liberated, young and mid microspores showed a spherical shape, being partially covered by the last pieces of the callosic layer (Figure 4D). Their exine exhibited a scabrate pattern characterized by presence of protrusions sticking out less than one micron from a layer full of deep cleavages regularly distributed along its surface (Figure 4D′). After that, in vacuolate microspore stage of development (Figure 4E) the triporate nature of the cannabis pollen grain was revealed. Throughout the exine, three prominent apertures were clearly visible. Each aperture (Figure 4E′) was composed by a circular pore whose diameter reached ≈1.0 μm. It was surrounded by a pore protrusion with a diameter of ≈3.5 μm, which raised up ≈0.8 μm from the exine. Following this phase, at young bi-cellular pollen stage of development (Figure 4F), some variation in the exine ornamentation was perceived. Mainly, the rifts responsible for the characteristic texture acquired by microspores during their development were not present. Instead of this, a layer resembling sporopollenin seemed to have partially filled the fissures, only keeping visible some protuberances emerging along the entire surface of the pollen grain (Figure 4F′). Finally, in mature pollen developmental stage (Figure 4G), a reduction in pore protrusion was observed. It decreased until the annular prominence circumscribing the pore was hardly distinguishable, while exine scabrate pattern was mainly composed by the tip of protuberances arising from a smooth layer of sporopollenin (Figure 4G′). Regarding exine evolution, no differences were observed neither among Finola and USO31 varieties, nor androecious, monoecious, and gynoecious plants.

### Development of the Different Layers That Compose the Anther Wall During Stamen Formation

**FIGURE 5:**
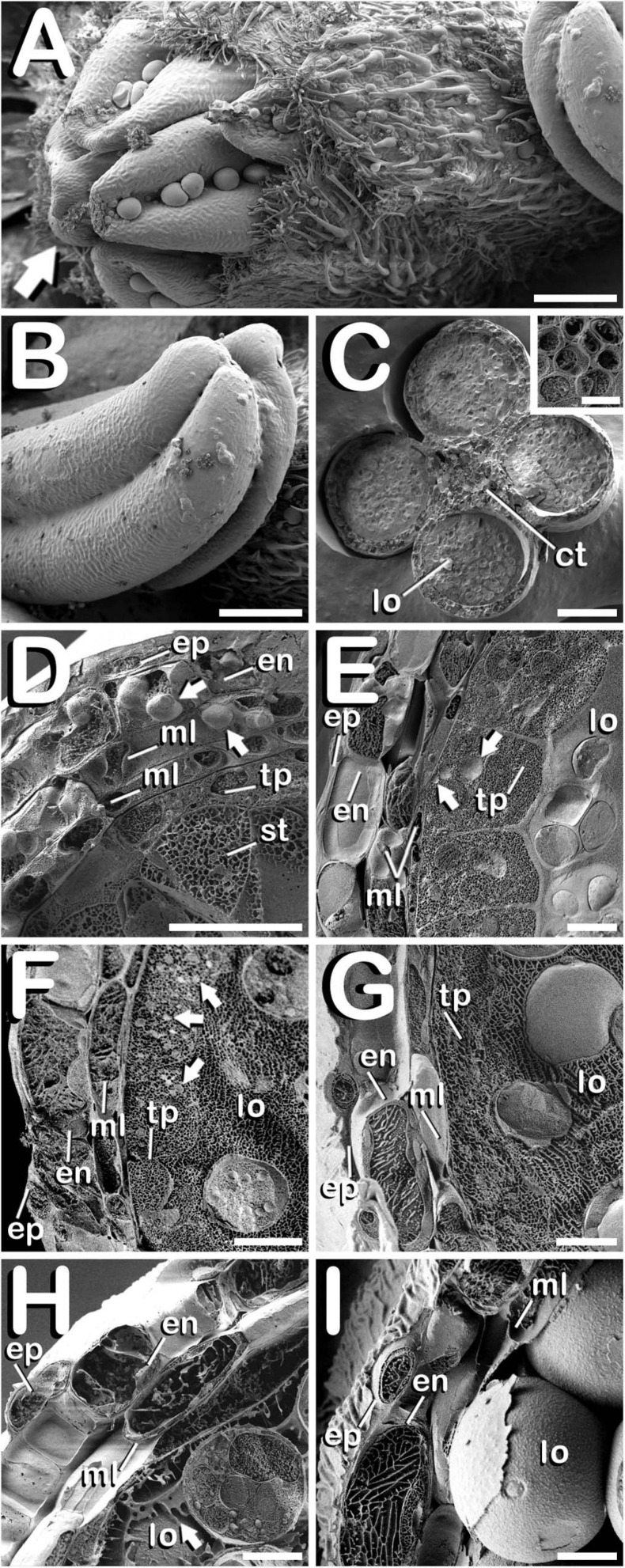
Cryo-SEM images showing anther wall development in *C. sativa*. The different developmental stages are described as follows: **(A)** Staminate flower with its five anthers being visible: arrow points to the anthers. **(B)** Anther excised from a male bud. **(C)** Cross-section of an anther after freeze-fracture showing four microsporangia and vascular bundles from connective tissue (inset in Figure 5C). **(D)** Microspore mother cell stage: arrows point plastids in endothecium and outer middle layer. **(E)** Tetrad stage: arrows point orbicules embedded in tapetum layer. **(F)** Young and mid microspore stage: arrows point orbicules embedded in tapetum layer. **(G)** Vacuolate microspore stage. **(H)** Young bi-cellular pollen stage: arrow points to tapetum sticky remnants covering the pollen grain surface. **(I)** Mature pollen stage. Scale bars **(A,B)**: 200 μm. Scale bars **(C)**: 100 μm. Scale bar (inset in **C**): 5 μm. Scale bars **(D–I)**: 10 μm. lo, locule; ct, connective tissue; ep, epidermis; en, endothecium; ml, middle layer; tp, tapetum; st, sporogenous tissue.

The study of fractured anthers through cryo-SEM, allowed for the elucidation of the changes developed in the different tissues that compose the anther wall during the whole process of pollen grain formation. Development of these layers was closely related with the stage of development of the microspores and pollen grains contained in the locule, which denotes a synchronized development between them. *Cannabis sativa* male buds contained five anthers (arrow in Figure 5A), which were individually excised (Figure 5B) in order to carry out freeze-fracture of the anthers (Figure 5C). After that, the four locules of each anther joined by the connective tissue, which was mainly composed by vascular bundles (inset in Figure 5C) were clearly visible. All the layers of the anther wall were already developed by the time of MMCs differentiation (Figure 5D). The anther wall was composed by an external epidermis followed by endothecium, two middle layers and tapetum. Endothecium and the outer middle layer presented a high number of rounded plastids ranging in size from 1 to 3 μm in diameter (arrows in Figure 5D). Total anther wall thickness at this stage was ≈17 μm. The anther wall layers enclosed the sporogenous tissue, which completely filled the loculus. After meiosis, in tetrad stage (Figure 5E), while epidermis and the inner middle layer were narrowed, tapetum layer reached its maximum thickness. Some bright orbicules of spherical shape embedded on tapetal cells were observed (arrows in Figure 5E). Their diameter oscillated between 1 and 5 μm. At this phase, total anther wall width extended to ≈45 μm. As microspores progressed in their development, degeneration of tapetum was more severe, being clearly visible from the young and mid-microspore stage of development (Figure 5F). At this stage, the inner middle layer was completely degraded and some fissures starting from the inner face of tapetal cells advanced perpendicularly to the outer layers of the anther wall, whose total thickness was reduced to ≈28 μm. Degeneracy of tapetal cells allowed observation of the orbicules enclosed in tapetum (arrows in Figure 5F), which in this stage ranged in size from 1 to 2 μm in diameter. Thereafter, in vacuolate microspore stage (Figure 5G), tapetum cells showed strong signs of degradation, being quite difficult to detect. At this phase, anther wall reached a total thickness of ≈24 μm, while in the young bi-cellular pollen stage of development (Figure 5H), the tapetal layer was absent, and in its place it was observed how tapetum sticky remnants covered the surface of pollen grains (arrow in Figure 5H), which were in direct contact with the remaining middle layer. In this developmental stage the anther wall reached ≈20 μm in some sections. Finally, the mature pollen stage (Figure 5I) was characterized by anther locule dehydration, which resulted necessary for subsequent anther longitudinal dehiscence through septum and stomium degeneration. There was no trace of the locular fluid and only mature pollen grains ready for dispersal were visualized. As in the previous phase, only epidermis, endothecium and middle layer were observed. Their total width was ≈15 μm, which was the minimal thickness of the anther wall measured in this study. No differences were observed for anther wall development either in Finola or USO31 varieties, or among androecious, monoecious, and gynoecious plants.

### Amyloplasts in Anthers, Microspores, and Pollen Grains From *C. sativa*

**FIGURE 6:**
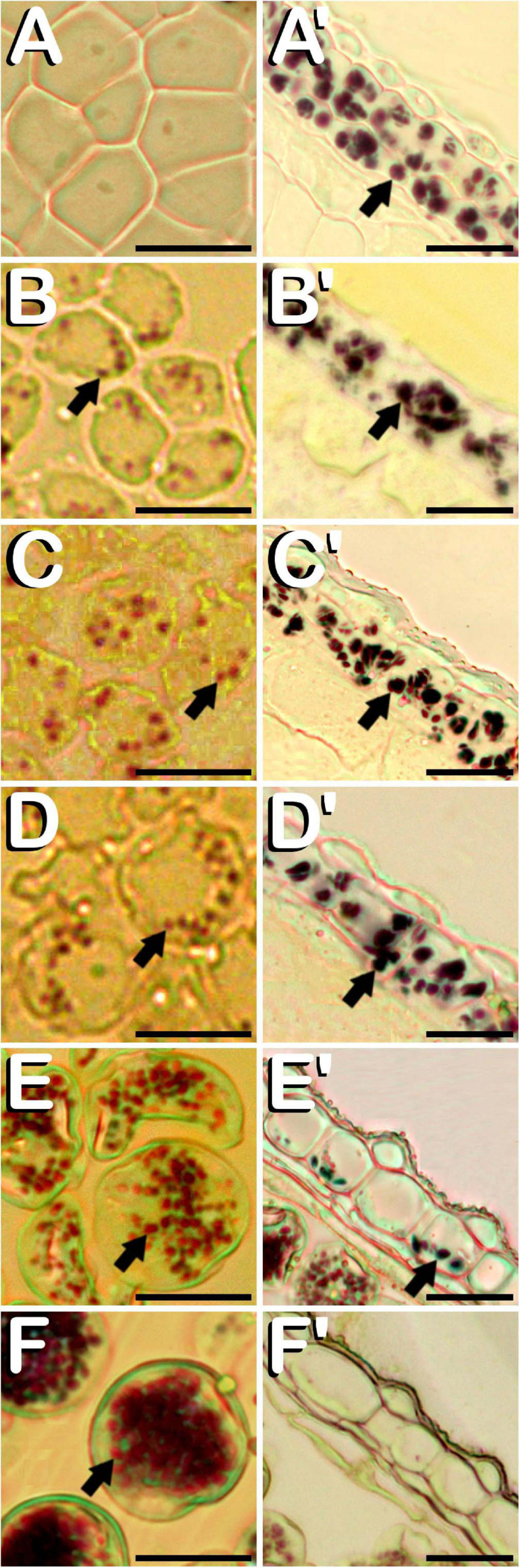
Histochemical detection of the amyloplasts contained in microspores and pollen grains **(A–F)**, and in endothecium and the outer middle layer of the anther wall **(A′–F′)** throughout microsporogenesis and microgametogenesis in *C. sativa*. The different developmental stages are described as follows: **(A,A′)** Microspore mother cell stage. **(B,B′)** Tetrad stage. **(C,C′)** Young and mid microspore stage. **(D,D′)** Vacuolate microspore stage. **(E,E′)** Young bi-cellular pollen stage. **(F,F′)** Mature pollen stage. **(A–F,A′–F′)** Bright-field microscope images after iodine-starch complex staining. Arrows point to amyloplasts. Scale bars **(A–F,A′–F′)**: 20 μm.

Amyloplasts were detected in all the different developmental stages of meiosis, microsporogenesis and microgametogenesis, although in meiotic stages of development, they were only present in some anther wall layers. Specifically, from MMC stage (Figure 6A) and throughout meiosis, starch deposits were only visualized in endothecium and the outer middle layer of the anther wall (Figure 6A′). Amyloplasts exhibited a dark purple coloration. MMCs lacked any kind of starch accumulation. After emergence of tetrads (Figure 6B), amyloplasts showing a red coloration started to be visible within the four microspores contained inside of the callose wall. At this stage, dark purple amyloplasts remained in endothecium and the outer middle layer of the anther wall (Figure 6B′). In young and mid microspore stages (Figure 6C), the number and coloration of the starch deposits contained inside of the microspores followed a similar trend as was observed for tetrad stage. Still in this stage, endothecium and the remaining middle layer showed carbohydrates reserves in the form of dark purple stained starch deposits (Figure 6C′). When microspores reached the vacuolate microspore stage and due to the space restrictions promoted by the development of a large vacuole, red stained microspore amyloplasts were exclusively located in the periphery of the microspore (Figure 6D), while dark purple amyloplasts were still present in endothecium and middle layer (Figure 6D′). After first pollen mitosis (Figure 6E), the amyloplast content of the pollen grains was increased. They were distributed along the entire pollen grain cytoplasm and presented a red to purple coloration. Simultaneously, energy reserves of the anther tissue were drastically reduced, with the last remnants of dark blue stained amyloplasts being present in endothecium (Figure 6E′). From this phase onward, no more amyloplasts were detected in the anther wall. The progressive increase of amylogenesis observed during pollen maturation concluded in mature pollen stage (Figure 6F). Pollen grains drastically increased their amyloplast content, appearing totally filled with purple stained amyloplasts. No energy reserves in the form of starch grains were observed in the remaining anther layers (Figure 6F′). It should be noted how tapetum layer did not present organelle-staining in none of the developmental stages studied.

### Correlation of the Bud Length With the Different *C. sativa* Microgametophyte Developmental Stages

A correlation between bud length and the different stages of microsporogenesis and microgametogenesis was observed in all phenotypes evaluated. Small buds showed earlier microspore stages of development and, as the buds grew, these stages disappeared and more advanced stages emerged. The Spearman rank correlation coefficient calculated was ρ = 0.9428, which denotes a strong positive correlation among bud length interval and pollen maturation. However, some differences with respect to the correlation of the bud length and the developmental stage of microspores and pollen grains contained were detected for the studied cultivars. The main difference observed was the fact that androecious plants from neutral-day variety Finola were the fastest maturing plants of this experiment, as male buds presented more advanced stages of development in less sized buds in comparison with the rest of evaluated phenotypes. Regarding the presence of the commonly considered as suitable stages for androgenesis induction in *C. sativa* male floral buds, significant differences were identified between the different bud length intervals established. The bud length interval which significantly presented the highest percentage of vacuolate microspores (70.00%) coexisting with young bi-cellular pollen grains (15.00%) was 2.00 to 2.99 mm (Table 2). With respect to gynoecious Finola plants, buds oscillating from 4.00 to 4.99 mm were found to contain significantly higher amounts of vacuolate microspores (68.42%) together with the lowest content of young bi-cellular pollen grains (5.26%) (Table 2).

**TABLE 2:** Correlation of bud length with meiosis, microsporogenesis, and microgametogenesis in different phenotypes of *C. sativa*.

					**Microspore and pollen grain developmental stages (%)**					
**Reproduction**	**Photoperiodism**	**Gender**	**Variety**	**Bud Length (mm)**	**MEIOSIS**	**TET**	**YM-MM**	**VM**	**YBP**	**MBP**
Dioecious	Neutral-Day	♂	Finola	1.00–1.99	40.90^a^ ± 10.73	31.82^a^ ± 10.16	22.73^a^ ± 9.14	4.55^b^ ± 4.55	0.00^c^ ± 0.00	0.00^b^ ± 0.00
				2.00–2.99	0.00^b^ ± 0.00	10.00^b^ ± 6.88	5.00^b^ ± 5.00	70.00^a^ ± 10.51	15.00^bc^ ± 8.19	0.00^b^ ± 0.00
				3.00–3.99	0.00^b^ ± 0.00	0.00^b^ ± 0.00	0.00^b^ ± 0.00	20.00^ab^ ± 9.18	55.00^ab^ ± 11.41	25.00^ab^ ± 9.93
				4.00–4.99	0.00^b^ ± 0.00	0.00^b^ ± 0.00	0.00^b^ ± 0.00	0.00^b^ ± 0.00	70.00^a^ ± 10.51	30.00^ab^ ± 10.51
				5.00–5.99	0.00^b^ ± 0.00	0.00^b^ ± 0.00	0.00^b^ ± 0.00	0.00^b^ ± 0.00	50.00^ab^ ± 8.01	50.00^a^ ± 8.01
Dioecious	Neutral-Day	♀ (STS)	Finola	1.00–1.99	100.0^a^ ± 0.00	0.00^b^ ± 0.00	0.00^b^ ± 0.00	0.00^b^ ± 0.00	0.00^b^ ± 0.00	0.00^b^ ± 0.00
				2.00–2.99	66.67^b^ ± 11.43	33.33^a^ ± 11.43	0.00^b^ ± 0.00	0.00^b^ ± 0.00	0.00^b^ ± 0.00	0.00^b^ ± 0.00
				3.00–3.99	0.00^c^ ± 0.00	16.67^ab^ ± 9.04	61.11^a^ ± 11.82	22.22^ab^ ± 10.08	0.00^b^ ± 0.00	0.00^b^ ± 0.00
				4.00–4.99	0.00^c^ ± 0.00	0.00^b^ ± 0.00	26.32^ab^ ± 10.38	68.42^a^ ± 10.96	5.26^b^ ± 5.26	0.00^b^ ± 0.00
				5.00–5.99	0.00^c^ ± 0.00	0.00^b^ ± 0.00	0.00^b^ ± 0.00	8.70^b^ ± 6.01	39.13^a^ ± 10.41	52.17^a^ ± 10.65
Dioecious	Short-Day	♂	USO31	1.00–1.99	100.0^a^ ± 0.00	0.00^b^ ± 0.00	0.00^b^ ± 0.00	0.00^b^ ± 0.00	0.00^b^ ± 0.00	0.00^a^ ± 0.00
				2.00–2.99	83.33^a^ ± 9.04	16.67^b^ ± 9.04	0.00^b^ ± 0.00	0.00^b^ ± 0.00	0.00^b^ ± 0.00	0.00^a^ ± 0.00
				3.00–3.99	10.53^b^ ± 7.23	52.63^a^ ± 11.77	31.58^a^ ± 10.96	5.26^b^ ± 5.26	0.00^b^ ± 0.00	0.00^a^ ± 0.00
				4.00–4.99	0.00^b^ ± 0.00	5.26^b^ ± 5.26	26.32^ab^ ± 10.38	68.42^a^ ± 10.96	0.00^b^ ± 0.00	0.00^a^ ± 0.00
				5.00–5.99	0.00^b^ ± 0.00	0.00^b^ ± 0.00	5.56^ab^ ± 5.56	88.89^a^ ± 7.62	5.56^b^ ± 5.56	0.00^a^ ± 0.00
				6.00–6.99	0.00^b^ ± 0.00	0.00^b^ ± 0.00	0.00^b^ ± 0.00	44.44^ab^ ± 12.05	44.44^a^ ± 12.05	11.11^a^ ± 7.62
Dioecious	Short-Day	♀ (STS)	USO31	1.00–1.99	100.0^a^ ± 0.00	0.00^b^ ± 0.00	0.00^b^ ± 0.00	0.00^b^ ± 0.00	0.00^b^ ± 0.00	0.00^b^ ± 0.00
				2.00–2.99	75.00^b^ ± 9.93	25.00^ab^ ± 9.93	0.00^b^ ± 0.00	0.00^b^ ± 0.00	0.00^b^ ± 0.00	0.00^b^ ± 0.00
				3.00–3.99	0.00^c^ ± 0.00	31.58^a^ ± 10.96	52.63^a^ ± 11.77	15.79^b^ ± 8.59	0.00^b^ ± 0.00	0.00^b^ ± 0.00
				4.00–4.99	0.00^c^ ± 0.00	0.00^b^ ± 0.00	0.00^b^ ± 0.00	95.24^a^ ± 4.76	4.76^b^ ± 4.76	0.00^b^ ± 0.00
				5.00–5.99	0.00^c^ ± 0.00	0.00^b^ ± 0.00	0.00^b^ ± 0.00	16.00^b^ ± 7.48	60.00^a^ ± 10.00	24.00^a^ ± 8.72
Monoecious	Short-Day	♂ + ♀	USO31	1.00–1.99	100.0^a^ ± 0.00	0.00^b^ ± 0.00	0.00^b^ ± 0.00	0.00^c^ ± 0.00	0.00^c^ ± 0.00	0.00^c^ ± 0.00
				2.00–2.99	75.86^b^ ± 8.09	13.79^b^ ± 6.52	10.34^b^ ± 5.76	0.00^c^ ± 0.00	0.00^c^ ± 0.00	0.00^c^ ± 0.00
				3.00–3.99	3.33^c^ ± 3.33	33.33^a^ ± 8.75	33.33^a^ ± 8.75	30.00^ab^ ± 8.51	0.00^c^ ± 0.00	0.00^c^ ± 0.00
				4.00–4.99	0.00^c^ ± 0.00	0.00^b^ ± 0.00	20.00^ab^ ± 7.43	46.67^a^ ± 9.26	20.00^b^ ± 7.43	13.33^bc^ ± 6.31
				5.00–5.99	0.00^c^ ± 0.00	0.00^b^ ± 0.00	0.00^b^ ± 0.00	40.00^a^ ± 10.00	36.00^ab^ ± 9.80	24.00^ab^ ± 8.72
				6.00–6.99	0.00^c^ ± 0.00	0.00^b^ ± 0.00	0.00^b^ ± 0.00	11.11^bc^ ± 7.62	50.00^a^ ± 12.13	38.89^a^ ± 11.82

*For each bud length interval (described in mm), at least 18 buds were used, and the mean of each developmental stage is expressed as a percentage (±SE) relative to the total sample size of the interval. Stages corresponding to microspore mother cell, meiocyte with two nuclei and meiocyte with four nuclei are merged together in a single meiotic stage of development. TET, tetrad; YM-MM, young and mid microspore; VM, vacuolate microspore; YBP, young bi-cellular pollen; MBP, mid bi-cellular pollen. Different letters among bud sizes in each genotype indicate significant differences between them (*p* < 0.05) according to non-parametric Kruskal–Wallis and pairwise Nemenyi tests. ♂: “Only male flowers present in the plant”. ♀: “Only female flowers present in the plant”. ♂ + ♀: “Male and female flowers present in the plant”.*

On the other hand, concerning short-day phenotypes, androecious USO31 plants had a significantly higher quantity of vacuolate microspores (88.89%) coexisting with young bi-cellular pollen grains (5.56%) in male buds fluctuating from 5.00 to 5.99 mm (Table 2), while the best bud length interval for gynoecious USO31 plants was 4.00 to 4.99 mm, which showed significant differences in comparison with the rest of bud length intervals evaluated and contained 95.24% of vacuolate microspores together with 4.76% of young bi-cellular pollen grains (Table 2). Regarding monoecious USO31 plants, the highest percentage of vacuolate microspores (46.67%) was found in buds ranging from 4.00 to 4.99 mm, which also contained microspores in young and mid microspore stage (20.00%), and pollen in young bi-cellular (20.00%) and mid bi-cellular pollen stage (13.33%) (Table 2). It is worth noting that the bud length interval from monoecious plants with more vacuolate microspores, showed the lowest percentage in comparison with the bud length intervals from the rest of phenotypes evaluated which contained the highest percentage of vacuolate microspores.

### Effect of Cold-Shock Bud Pretreatment on Microspore Viability, Amyloplast Content, and Development of Multicellular Structures of Androgenic Origin

After merging together data from all evaluated phenotypes, it was found that the cold-shock applied on buds during 1 week resulted in a statistically significant reduction of microspore viability, which fell from 62.13% of viable microspores and pollen grains under *in vivo* conditions to 46.89% of viability rate for cold treated buds (Table 3). Despite of this, some of the evaluated phenotypes did not show a significant reduction of microspore viability, as is the case of androecious and gynoecious plants from Finola (Table 3). On the other hand, amyloplast content of vacuolate microspores and young bi-cellular pollen grains did not vary after exposure of male buds to a week-long cold-shock at 4°C ± 1°C, showing the same starch distribution pattern as observed under *in vivo* conditions.

**TABLE 3:** Effect of a week-long cold pretreatment (4°C ± 1°C) applied directly on excised buds from *C. sativa* prior to microspore *in vitro* culture.

**Treatment**	**All varieties**	**♂ Finola**	**♀ (STS) Finola**	**♂ USO31**	**♀ (STS) USO31**	**♂ + ♀ USO31**
Microspore and pollen grains from flower buds (*in vivo*)	62.13^a^ ± 2.11	53.71^a^ ± 4.61	59.71^a^ ± 4.07	70.88^a^ ± 4.27	60.49^a^ ± 1.45	65.87^a^ ± 3.64
Microspores from excised flower buds at 4°C during 7 days	46.89^b^ ± 2.53	45.61^a^ ± 3.05	50.35^a^ ± 6.38	42.36^b^ ± 9.04	52.30^a^ ± 5.22	43.81^b^ ± 5.27

*Mean of microspore viability rates is expressed as a percentage (±SE) calculated from data coming from at least three replicates. Different letters among treatments indicate significant differences between them (*p* < 0.05) according to parametric ANOVA and pairwise Fisher’s least significant difference (LSD) tests. ♂: “Only male flowers present in the plant”. ♀: “Only female flowers present in the plant”. ♂ + ♀: “Male and female flowers present in the plant”.*

**FIGURE 7:**
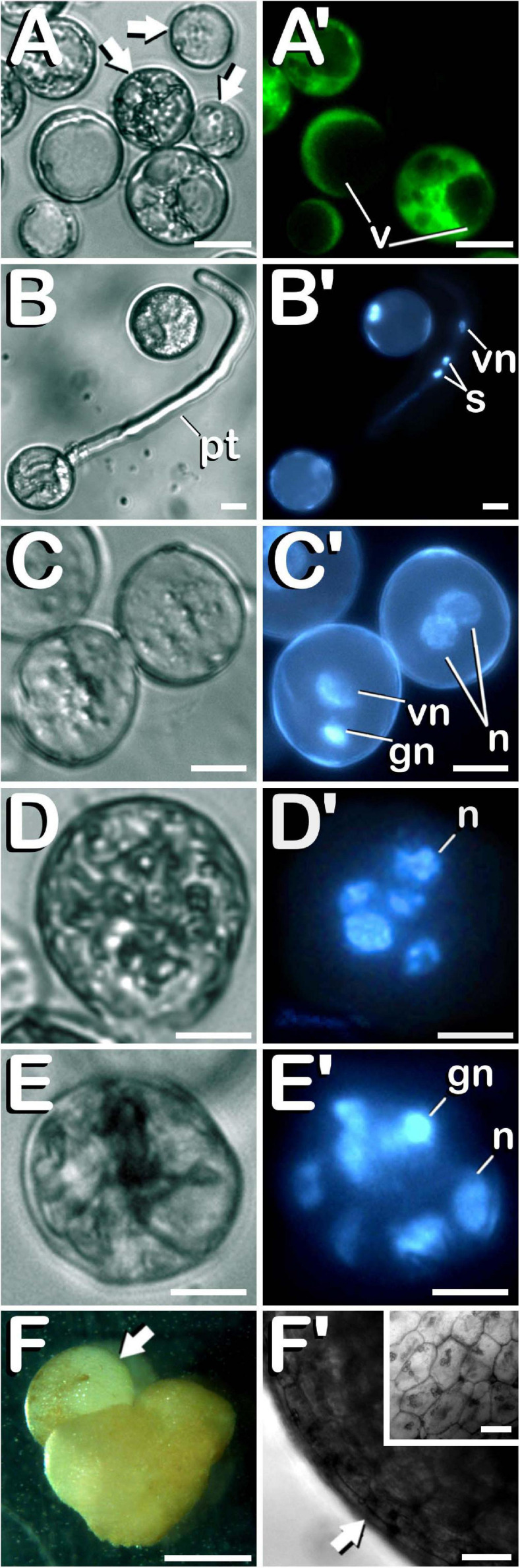
Characterization of the different developmental pathways of *C. sativa* microspores and pollen grains cultured under *in vitro* conditions. The different developmental pathways are described as follows: **(A,A′)** An enriched population of vacuolate microspores and young bi-cellular pollen grains just after *in vitro* culture stablishment: Arrows point to dead microspores, which does not exhibit any fluorescence. **(B,B′)** Gametophytic pathway developed by a pollen grain culminated with the emission of a prominent pollinic tube. **(C,C′)** Illustration of both, gametophytic and sporophytic pathways developed by microspores after a week-long cold-shock bud pretreatment. **(D,D′)** Microspore under sporophytic development showing five nuclei as a result of successive divisions promoted by cold-shock bud pretreatment. **(E,E′)** Pollen-derived embryogenic structure generated after cold-shock bud pretreatment and three weeks of *in vitro* culture. **(F)** First *C. sativa* microspore-derived embryos: Illustration of a secondary embryogenesis event which resulted in the development of a globular embryo on a heart-shaped microspore-derived embryo: arrow points to the globular embryo. **(F′)** Protoderm development (arrow) in the embryogenic structure, which showed a well-organized and regular cellular structure (inset in **F′**). **(A–E)** Differential Interference Contrast (DIC) microscope images. **(A′)** Fluorescent microscope image after FDA staining. **(B′–E′)** Fluorescent microscope images after DAPI staining. **(F)** Stereomicroscope image. **(F′)** Bright-field microscope image. Scale bars **(A–E,A′–E′)**: 10 μm. Scale bar **(F)**: 1 mm. Scale bars **(F′)** and (inset in **F′**): 50 μm. v, vacuoles; pt, pollinic tube; vn, vegetative nucleus; s, spermatids; gn, generative nucleus; n, sporophytic nucleus.

Finally, different cannabis microspore developmental pathways were observed under *in vitro* conditions. Some of the microspores and pollen grains coming from both non-pretreated and cold-pretreated buds were already dead when starting *in vitro* culture (arrows in Figure 7A), as shown by the lack of enzymatic activity and/or cell-membrane integrity and its consequent absence of fluorescence (Figure 7A′). It is worth mentioning that FDA vital staining did not penetrate into vacuoles, retaining its fluorescent product in the cytoplasm of viable vacuolate microspores and young bi-cellular pollen grains. Other microspores and pollen grains followed their gametophytic developmental pathway with the consequent germination of the pollinic tube (Figure 7B), through which spermatids circulated behind the vegetative nucleus (Figure 7B′) trying to carry out fertilization of endosperm and egg cell. On the other hand, and although with an extremely low frequency (only four microspore-derived embryogenic structures were observed in our experiments), it should be noted how embryogenic microspores were also observed, but only after cold pretreatment of flower buds. As a consequence of cold-shock bud pretreatment prior to *in vitro* culture, some microspores and pollen grains coming from androecious and gynoecious USO31 plants deviated from their gametophytic pathway toward a sporophytic development. The first signal of microspore reprogramming was observed just after cold-shock bud pretreatment, which resulted in a striking nuclear disturbance. Instead of generating a vegetative nucleus and a more condensed generative nucleus as a result of first pollen mitosis, a sporophytic microspore from a gynoecious USO31 plant showed two nuclei similar in size and degree of chromatin condensation (Figures 7C,C′). Furthermore, just after exposure of buds to a week-long cold-shock, another multicellular microspore from the previously mentioned replicate containing up to 5 nuclei was also observed (Figures 7D,D′), while 3 weeks after *in vitro* culture, an embryogenic pollen grain from an androecious USO31 plant containing 7≈8 nuclei was found (Figures 7E,E′). It is necessary to highlight how, in this last case, among the different nuclei observed, one of them seemed to be a generative nucleus. While previous described events constituted the first stages of *Cannabis* microspore embryogenesis, 9 weeks after *in vitro* culture a microspore-derived embryo (Figure 7F) from the previously mentioned androecious USO31 replicate was identified. Specifically, a heart-shaped embryo which resembled to have suffered a secondary-embryogenesis event that promoted development of a globular embryo on it was visualized (arrow in Figure 7F). The embryogenic structure developed protoderm (arrow in Figure 7F′) and showed a well-organized and regular cellular structure (inset in Figure 7F′). During its development, this embryogenic structure showed oxidation symptoms, as reflected by browning, stopping its development from the moment of its visualization onward. In summary, both gynoecious and androecious USO31 phenotypes produced 1.11 microspore-derived embryoids per 100 anthers (each phenotype produced two microspore-derived multicellular structures from 180 anthers squashed to perform the microspore culture).

## Discussion

### *Cannabis sativa* Is a Suitable Candidate for Androgenesis Experiments Mainly Due to Its Fast Production of Viable and Potentially Inducible Microspores and Pollen Grains

Even though the various phenotypes evaluated showed different sexual systems (dioecious and monoecious), photoperiodism (short and neutral-day) and sex (male and female), direct exposition of seedlings to a photoperiod constituted by 12 h of light per day resulted in male bud availability in all evaluated phenotypes just 30 days after seed germination. As observed in other *C. sativa* works, male plants were taller than female individuals (Onofri and Mandolino, 2017). The great amount of microspores and pollen grains produced in each flower bud (>150,000), together with the high rates of microspore viability reached for all phenotypes (>50%), makes *C. sativa* a suitable system able to provide, in a very quick period of time (≈30 days after seed germination), huge populations of viable vacuolate microspores and young bi-cellular pollen grains potentially inducible to afford microspore and pollen embryogenesis experiments. Viability rates obtained in our research are similar to the ones reported by Choudhary et al. (2014) which, depending on the season, oscillated among 33.3 and 83.0%, while in another work (Zottini et al., 1997) a viability rate for mature pollen grains of 92% was reported. This difference among viability rates could be attributable to the microspore isolation procedure, as viability measurements in the former works were performed on mature pollen grains naturally released after anthesis. Regarding gynoecious phenotypes, undoubtedly the most interesting of the species due to their exclusive capability for secondary metabolite production (Small, 2017), it is worth noting how sexual reversion did not influence microspore viability, as compared to androecious and monoecious specimens. This particularity allows to assay microspore embryogenesis protocols with gynoecious plants, which could lead to produce the first cannabis female pure lines 100% homozygous in only one *in vitro* generation. These double haploids could be used in breeding programs for the development of authentic high-yielding female hybrids, whose biochemical profile could be reliably reproduced through seed. Moreover, taking into account the already known XX female sexual chromosomal inheritance of the species (Hirata, 1927; Faux et al., 2014), and how promotion of male flowers on cannabis female plants can be routinely achieved through STS treatment (Ram and Sett, 1982), it would also be possible to self-pollinate the double haploids obtained, thus keeping these genotypes through seed and avoiding the perpetual maintenance of mother plants through vegetative propagation.

### Anthers From *C. sativa* Present a High Degree of Uniformity in the Developmental Stages of Microspores and Pollen Grains Contained

Despite of their biological differences, none of evaluated phenotypes showed significant differences between them throughout the different developmental stages emerged during microsporogenesis and microgametogenesis. Additionally, a high uniformity grade was observed both in the developmental stage of microspores and pollen grains contained in anthers, as well as among anthers coming from the same bud, which presented microspores and pollen grains in the same stage of development. In all phenotypes, MMCs entered in meiosis simultaneously. This synchronized development could be attributable to the presence of cytoplasmic connections between meiocytes, as it has been described in *C. sativa* (Heslop-Harrison, 1966; Mascarenhas, 1975). On the other hand, the present work also certifies the cytokinesis by furrowing already described in this species (Reed, 1914; McPhee, 1924), evidenced by the coexistence of different nuclei in the same cytoplasm after meiosis I and meiosis II, and the polygonal shape of the microspores enclosed by the callosic layer in tetrad stage. Size of mature pollen grains observed in this study was similar to the data published in other works (Punt and Malotaux, 1984; French and Moore, 1986; Shinwari et al., 2015; Halbritter, 2016), which indicates that the conditions tested in our experiments did not affect the size of mature pollen grains. With respect to the second pollen mitosis developed in this species, and in contrast with results published in other *C. sativa* related works (Asanova, 2002), this study demonstrates that it can occur before germination of the pollen tube, as it has been reported in other species such as *Arabidopsis thaliana* L. Heynh. or *Zea mays* L. (Ma, 2005).

Regarding identification of vacuolate microspores and young bi-cellular pollen grain stages of development in *C. sativa*, it should be noted that fluorescence microscopy has been shown to be the most effective technique to discriminate among the different microspore and pollen grain stages of development in this species. This technique allowed a detailed study of nuclear dynamics emerged during the whole process of pollen grain formation, which finally represent the most reliable and easily identifiable events associated with the different developmental stages studied.

### The Exine Ornamentation of *C. sativa* Microspores and Pollen Grains Presents a Scabrate Sculpture Which Is Almost Completely Covered by Sporopollenin at Maturity

After degradation of the callose wall, young and mid microspores appeared spherical and showed their scabrate sculpture, which was slightly modified through progressive addition of sporopollenin on its surface until maturity. It may be noted how sporopollenin deposition and its role in exine pattern and pollen wall formation is preserved across taxa (Wiermann and Gubatz, 1992; Ariizumi and Toriyama, 2011; Borg and Twell, 2011; García et al., 2017). As protrusions observed during microspore development did not stick out more than a micron from the pollen surface in any of its developmental stages, its exine sculpture perfectly fits the scabrate pattern described by Laín (2004). Thus, although our results are in contrast with those reported by Halbritter (2016), who defined the exine of cannabis mature pollen grain as granulate, previously published works are in line with our findings (Bradley, 1958; Punt and Malotaux, 1984; Shinwari et al., 2015). Despite the robust exine exhibited by microspores and pollen grains of *C. sativa*, it could be traversed by microchannels that, together with apertures, could act as routes for nutrient uptake from loculus into pollen cytoplasm, as has been described in other species like *Olea europaea* L. (Fernández and García, 1990), *Betula verrucosa* Ehrh. and *Chenopodium album* Bosc ex Moq (Rowley et al., 1987), or *Lopezia*, *Gaura*, and *Gelsemium* (Rowley et al., 2003). The fact that microspores and pollen grains of *C. sativa* are surrounded by locular fluid for nutrient supply during great part of their development, coupled with the possible existence of these microchannels distributed in the exine, could also explain the high homogeneity observed in the stage of development of microspores and pollen grains contained in the microsporangium. The fact that desynchronization in development was only detected in mature pollen stage, when mid bi-cellular and mature tri-cellular pollen grains coexisted in the microsporangium (which lacked locular fluid), could confirm this hypothesis.

### The Anther Wall of *C. sativa* Is Composed by an External Epidermis, Endothecium, Two Middle Layers and a Secretory Type of Tapetum

Anther wall composition of *C. sativa* corresponds with the typical pattern commonly described in angiosperms (Clément and Pacini, 2001). In addition, Reed (1914) described the same wall layers in his observations concerning cannabis stamen formation. However, some discordances among results reported by Reed (1914) and our results were detected. Specifically, while in the former work it is described how inner middle layer had already disappeared in tetrad stage, and how only epidermis and endothecium persisted after locule dehydration, we observed remnants of the inner middle layer in tetrad stage and how rests of the outer middle layer were present in mature pollen stage of development. It is necessary to emphasize that in our study, anther dehydration took place at mature pollen stage, in which pollen grains were ready for their dispersal. In this respect, regarding locular fluid desiccation and depending on the species, it could be performed by passive transpiration through anther wall tissues, by reabsorption through stamen filament, or both, being greatly influenced by environmental factors (Pacini, 1994; Keijzer, 1999). It can be concluded that drastic reduction of the anther wall during pollen grain formation and maturation, together with anther dehydration, facilitates longitudinal dehiscence of the anther and subsequent pollen dispersal through the wind, main vector responsible for pollination in an anemophilous species such as *C. sativa*.

On the other side, it is important to highlight how tapetal cells must be considered essential for the reproductive status of angiosperms, being directly involved in crucial events such as callase supply, which digests the callose wall that encloses microspores in tetrad stage (García et al., 2017), nutrition of the microspores and pollen grains developed in the locule, and formation of the exine of mature pollen grains (Heslop-Harrison, 1962; Clément and Pacini, 2001; García et al., 2017). In our work, we observed small spheres embedded in the tapetum cell layer from tetrad until young and mid microspore stages of development. With regards to the function of these spheres, which have been previously described in *C. sativa*, some researchers argued that these orbicules could be derived from mitochondria (Heslop-Harrison, 1962), and that during tapetum cell degeneration, they could transport sporopollenin to the microspore exine, a complex of fatty acid derivatives and phenylpropanoids that form an extremely inert biopolymer in the exine to resist physical, biological and chemical attacks (Wang et al., 2003; Ariizumi and Toriyama, 2011). Huysmans et al. (1998) stated that, although there are some exceptions, presence of orbicules in the tapetal cells can be considered a general feature of a secretory type of tapetum, classifying these structures as Ubisch bodies. Moreover, in the same study, the author categorized the tapetum cell layer as secretory type when it remains *in situ* until its degeneration. With respect to their degeneration, it is well known how tapetal cells of secretory type degenerate by programmed cell death, process generally completed around the first microspore haploid mitosis (Ma, 2005; García et al., 2017), as it was found in the studied phenotypes. Thus, findings from our research support the idea of a secretory type of tapetum in *C. sativa*.

### Starch Content of Microspores and Pollen Grains From *C. sativa* Is Coincident With the Amyloplast Pattern Observed in Species Recalcitrant to Androgenesis

It was found that microspores and pollen grains from *C. sativa* contained amyloplasts from tetrad stage until mature pollen stage. During maturation, microspores and pollen grains experimented a progressive starch accumulation (especially after first pollen mitosis), along with the emptying of the starch reserves from the anther wall. Some authors have suggested how locule external surrounding layers (in particular their plastids) are mainly involved in sugar physiology, storing carbohydrates in form of starch grains (Clément and Audran, 1999; Clément and Pacini, 2001). It is necessary to highlight how our observations strongly suggest that carbohydrates reserves could be transported from microsporangium external surrounding layers into the locule for microspore absorption, as has already been described by other researchers (Bhandari, 1984; Keijzer and Willemse, 1988; Heberle-Bors, 1989; Clément and Pacini, 2001). On the other hand, total absence of amyloplast-staining in tapetum cells during microsporogenesis and microgametogenesis could imply that tapetal cells did not synthetize neither accumulate starch. Instead, other authors have reported that, in anemophilous species, tapetal plastids evolve into elaoiplasts, being responsible for lipid synthesis, accumulation and subsequent secretion into the locule (Clément and Pacini, 2001).

With regards to amyloplast coloration after iodine-starch complex staining, it has been reported how binding of iodine with amylose leads to the formation of deep-blue complexes, while binding with amylopectin results in a reddish-brown color formation, and how when hydrolyzed, both polysaccharides, as far as their chain length is reduced, gradually lose the capacity to be stained with iodine (Bates et al., 1943; Bailey and Whelan, 1961). The physical properties of starch, which is mainly conformed by two types of molecules namely amylose and amylopectin, could explain the different colors showed by amyloplasts in our experiments.

Finally, the pattern of plastids contained in microspores and pollen grains from *C. sativa* could help to estimate the androgenic potential of this species. Our observations fit perfectly with the description reported by Sangwan and Sangwan-Norreel (1987) for recalcitrant or non-androgenic species. As it can be concluded from our results, presence of amyloplasts from tetrad until mature pollen stage of development, along with the strong increase of starch biosynthesis in young bi-cellular pollen stage, points to a high level of recalcitrance of *C. sativa* to androgenic induction.

### The Bud Length Can Be Used as a Floral Morphological Marker to Identify Male Floral Buds Containing Specific Microgametophyte Developmental Stages

With respect to male bud maturity rate and the slight discrepancies observed among androecious plants from Finola and the rest of evaluated phenotypes, it should be noted how differences observed could be attributable to the fact that neutral-day Finola presented the early blooming character typical from ssp. *ruderalis* (Callaway and Laakkonen, 1996). As *C. sativa* plants belonging to ssp. ruderalis do not depend on a decrease of light hours in order to start flowering, they have a shorter life cycle than short-day varieties. Particularly, male plants show a faster development which drives them to enter sooner in senescence, which would force microspores to mature sooner to be able to carry out fertilization and continue with species evolution.

On the other hand, since stage of development of microspores and pollen grains is crucial for microspore embryogenesis (Ferrie et al., 1995; Dunwell, 2010; Canonge et al., 2020), establishment of the correlation between a simple, accurate and reproducible floral morphological marker and the different microgametophyte stages of development, must be considered as the first approach in order to study androgenesis induction in a species whose microspores have never been subjected to experimental embryogenesis, as is the case of *C. sativa*, thus increasing the population of specific developmental stages available for the induction. This approach has been successfully applied in model species in which a reliable and highly effective androgenesis induction protocol has been established like *Nicotiana tabacum* L. (Kasperbauer and Wilson, 1979) or *Oryza sativa* L. (Mishra and Rao, 2016). With this aim, this study was focused on finding bud length intervals that contained exclusively the commonly considered as suitable for androgenesis induction vacuolate microspores and young bi-cellular pollen grains. Additionally, an attempt was made to identify the bud length interval where young bi-cellular pollen stage started to appear. In this way, more vacuolate microspores close to go through first pollen mitosis can be present in the interval. If it was not possible and not only vacuolate microspores and young bi-cellular pollen grains were present in the interval, we recommend to choose the bud length interval with these developmental stages coexisting with earlier stages of development, because considering that microspores follow their development during *in vitro* culture, more microspores potentially inducible could be present in the buds. However, it should be noted that in our study, buds with the same length and coming from the same plant, do not necessarily contained microspores and pollen grains in the same developmental stages, as it occurred with buds of the same length belonging to different plants of the same variety. Thus, prior to routinely implementation, it is recommended to adapt this approach to each of the phenotypes with which experiments are going to be developed. Finally, due the absence of studies concerning microspore and pollen embryogenesis in *C. sativa*, we strongly encourage to study and evaluate the androgenic competence of all the microspore and pollen grain stages of development before consider that any of them is the most suitable for androgenesis induction in this species.

In light of the results obtained in this work, bud length is presented as a simple, accurate and reproducible floral morphological marker correlated with the stage of development of the microspores and pollen grains contained, although this method needs to be adjusted for each variety studied to get reliable and reproducible results. Through this methodology, it is possible to avoid additional work like the dissection of buds and the staining of the microspores and pollen grains with DAPI prior to *in vitro* culture for identifying their developmental stage, thus significantly increasing the number of potentially inducible microspores and pollen grains available for androgenesis induction experiments.

### Although With an Extremely Low Frequency, Cold-Shock Bud Pretreatment Can Promote Microspore Embryogenesis in *C. sativa*

Our research sheds light on the different developmental pathways of *C. sativa* microspores and pollen grains cultured under *in vitro* conditions, and how, although with an extremely low frequency, cold-shock pretreatment applied on buds can deviate the naturally occurring gametophytic pathway toward an embryogenic development. As a result of a week-long cold pretreatment applied directly on excised buds prior to microspore culture, a stress-derived slight decrease in microspore viability was detected. Our findings are in agreement with results published by Choudhary et al. (2014), who studied how pollen viability was significantly influenced by seasonal fluctuations in temperature and humidity. Researchers reported a significant decrease of pollen viability at low temperatures, reaching the lowest viability rates in the winter season. Interestingly, in our work, none of the Finola phenotypes evaluated (neither androecious nor gynoecious plants) showed a significant reduction of microspore viability rate after cold-shock bud pretreatment. This could be explained by the fact that Finola is an early blooming and frost tolerant hybrid developed in Finland (where genetic-selection work was conducted) and derived from Vavilov Research Institute (VIR) accessions descended from *C. sativa* ssp. *ruderalis*, which is thought native to the Altai region of Siberia (Callaway and Laakkonen, 1996). Furthermore, and although cold treatment has been found to partially or completely inhibit the formation of starch grains in *Datura* proplastids and in pollen from *Hordeum vulgare* L. (Sangwan and Sangwan-Norreel, 1987), this was not observed in vacuolate microspores and young bi-cellular pollen grains coming from a week-long cold pretreated *C. sativa* buds, whose starch distribution pattern was similar to the one observed under *in vivo* conditions.

On the other hand, our research constitutes the first illustration of the early events of microspore embryogenesis ontogeny in *C. sativa*. It is worth noting how analysis of nuclear dynamics through fluorescence microscopy proved to be the best option for unequivocal differentiation among embryogenic and gametophytic development of microspores, being also a useful tool for avoiding wrong diagnoses of different structures which could be incorrectly classified as embryos and/or multicellular structures of microspore origin (Bal et al., 2012). This represents a common problem in microspore embryogenesis research, specially with new species which have never been submitted to androgenesis induction experiments. First trace of microspore reprogramming was observed just after the week-long cold-shock bud pretreatment, consisting in the presence of two nuclei with a similar size and low-condensed chromatin. It has been suggested that first symmetrical division of the microspore leads to microspore embryogenesis in model species such as *Brassica napus* L. (Zaki and Dickinson, 1991) or *Nicotiana tabacum* L. (Nitsch, 1974), and also in recalcitrant species like *Antirrhinum majus* L. (Barinova et al., 2004), *Solanum lycopersicum* L. (Bal and Abak, 2005), or *Solanum melongena* L. (Bal et al., 2009). Furthermore, two multicellular embryogenic microspores containing several nuclei were also observed, although in one of them, the nuclei coexisted with a generative nucleus, which denotes that in this last case, successive divisions of the vegetative nuclei after first pollen mitosis gave rise to this embryogenic structure. This developmental pathway has already been related to microspore embryogenesis and described in model species such as *Nicotiana tabacum* (Sunderland and Wicks, 1971), or *Datura metel* L. (Iyer and Raina, 1972), and also in less studied species like *Solanum surattense* Burm. fil. or *Luffa cylindrica* L. Roem (Sinha et al., 1978). In this respect, it is worth noting that development of *C. sativa* pollen under low temperatures derived in nuclear disturbances observed during the course of meiosis, as was previously reported by Medwedewa (1935). Finally, more advanced stages of microspore embryogenesis were also identified in our study. Although it suffered a secondary embryogenesis event, showed oxidation signs and stopped its development, a microspore-derived embryo with a compact and regularly distributed cell structure, together with a well-differentiated protoderm was observed. It should be noted that formation of the protoderm is considered a marker for embryo formation (Yeung et al., 1996; Soriano et al., 2013). On the other hand, as it has been observed in our work, and as stated by other researchers (Fan et al., 1988; Telmer et al., 1995), the majority of sporophytic structures stop growing after a few divisions and die, which could explain why only multinuclear structures, which did not develop further, were observed at low frequency. In this respect it is necessary to emphasize that frequency of embryo induction and plant regeneration, together with secondary embryogenesis of the embryoids, are among some of the obstacles for an efficient implementation of microspore culture (Zhou et al., 2002). Finally, addition of auxins to the culture medium (Nehlin et al., 1995), failure to transfer embryos at the right time to regeneration or germination media (Oleszczuk et al., 2014), or the effect of culture temperature (Huang et al., 1991), could be responsible for secondary embryogenesis and growth arrest observed in our work.

## Conclusion

We have found that *C. sativa* is an appropriate candidate to be submitted to microspore and pollen embryogenesis experiments. The high amount of viable microspores and pollen grains present in its buds, together with the high uniformity grade observed in both the developmental stage of microspores and pollen grains contained in anthers, as well as among anthers coming from the same bud, and the fact that it was possible to correlate the different stages of development with bud length, represent important advantages that can be exploited by researchers who want to advance in this field. However, we recommend to adjust this method for each variety studied to get reliable and reproducible results. Furthermore, although the starch content of *C. sativa* microspores and pollen grains is coincident with the amyloplast pattern observed in species recalcitrant to androgenesis induction, here we demonstrate that it may be possible to deviate the naturally occurring gametophytic pathway toward an embryogenic development through cold-shock bud pretreatment. Although further studies are needed in order to optimize a reliable and effective protocol, here we lay the foundations for androgenesis research in this species, and propose this technique as a promising alternative for genetic standardization of *C. sativa* traits such as cannabinoid content of female inflorescences, avoiding the intrinsic heterogeneity of the species and setting the standards for the future of industrial and medical cannabis.

## Data Availability Statement

The datasets generated for this study are available on reasonable request to the corresponding author.

## Author Contributions

AG-Á, EG-F, JP, and FH conceived and designed the research. AG-Á and EG-F performed the experiments. AG-Á, EG-F, and FH analyzed the results. AG-Á wrote the manuscript. JP and FH reviewed and edited the manuscript. All authors have read and approved the manuscript for publication.

## Conflict of Interest

AG-Á was employed by company Ploidy and Genomics Ltd. The remaining authors declare that the research was conducted in the absence of any commercial or financial relationships that could be construed as a potential conflict of interest.
